# Insights into prognosis and immune infiltration of cuproptosis-related genes in breast cancer

**DOI:** 10.3389/fimmu.2022.1054305

**Published:** 2022-11-28

**Authors:** Tingting Huang, Yankuo Liu, Jiwei Li, Bingbing Shi, Zhengda Shan, Zhiyuan Shi, Zhangru Yang

**Affiliations:** ^1^ School of Medicine, Xiamen University, Xiamen, China; ^2^ Department of Critical Care Medicine, The Affiliated Hospital of Putian University, Putian, China; ^3^ School of Medicine, Sun Yat-Sen University, Shenzhen, China; ^4^ Department of Radiation Oncology, Shanghai Chest Hospital, Shanghai Jiao Tong University, Shanghai, China

**Keywords:** cuproptosis, breast cancer, PDHA1, prognosis, immune infiltration

## Abstract

**Introduction:**

Breast cancer (BC) has been ranking first in incidence and the leading cause of death among female cancers worldwide based on the latest report. Regulated cell death (RCD) plays a significant role in tumor initiation and provides an important target of cancer treatment. Cuproptosis, a novel form of RCD, is ignited by mitochondrial stress, particularly the lipoylated mitochondrial enzymes aggregation. However, the role of cuproptosis-related genes (CRGs) in tumor generation and progression remains unclear.

**Methods:**

In this study, the mRNA expression data of CRGs in BC and normal breast tissue were extracted from TCGA database, and protein expression patterns of these CRGs were analyzed using UALCAN. The prognostic values of CRGs in BC were explored by using KaplanMeier plotter and Cox regression analysis. Genetic mutations profiles were evaluated using the cBioPortal database. Meanwhile, we utilized CIBERSORT and TIMER 2.0 database to perform the correlation analysis between CRGs and immune cell infiltration.

**Results:**

Our results indicated that CRGs expression is significantly different in BC and normal breast tissues. Then we found that upregulated PDHA1 expression was associated with worse endpoint of BC. Moreover, we also performed immune infiltration analysis of CRGs, and demonstrated that PDHA1 expression was closely related to the infiltration levels of CD4+ memory T cell, macrophage M0 and M1 cell and mast cell in BC.

**Conclusions:**

Our results demonstrated the prognostic and immunogenetic values of PDHA1 in BC. Therefore, PDHA1 can be an independent prognostic biomarker and potential target for immunotherapy of BC.

## Introduction

Breast cancer is the most frequent and commonly diagnosed cancer in women, and ranks first in terms of morbidity and mortality (1, 2). Not only a wealth of examinations including breast ultrasound, mammography and MRI have been conducted, which can diagnose breast cancer patients in early stage (3), but breast cancer has evolved from classical markers providing basic tissue diagnosis, such as ER and HER2, to a series of comprehensive biomarkers including BRCA1/2, PIK3CA, FOXA1 and NAT1 based on protein expression and molecular prognosis (4–6). However, breast cancer is a highly heterogeneous disease with non-specific and complicated biomarkers. Furthermore, in recent years a growing body of research has revealed that about 20% of metastatic BC patients survive less than 5 years, which may be due to the lack of specific biomarkers for early diagnosis and the prognosis assessment of BC patients (7, 8). Thus, it is imperative to find effective biomarkers to assess the prognosis of BC patients and explore new thoughts for BC treatment.

Breast cancer has traditionally been considered as a limited immunogenic tumor, but now there is growing evidence that immune infiltration has a prognostic role in all breast cancer subtypes (9, 10). The number and composition of tumor-infiltrating lymphocytes (TILs) are critical for both breast cancer treatment responsiveness and improved prognosis. TILs comprise a mixture of cytotoxic T cells, helper T cells, B cells, macrophages, natural killer cells, and dendritic cells, which have been observed in many solid tumors, including breast invasive carcinoma(BRCA) (11), gastrointestinal tumor (12) and colorectal cancer (13). To date, robust immune biomarkers for therapy have not been established in BC. A comprehensive understanding of the association between gene expression in BC and tumor immune components may facilitate faster identification of novel immune-related targets and elucidation of the immune-mediated interaction in BC patients.

Copper plays an indispensable role in cells, which is a catalytic cofactor involved in the regulation of energy generation, iron collection, oxygen transport, signal transduction and plenty of other biological processes (14). Slight changes of copper homeostasis might generate severe toxicity and influence the initiation and progression process of cancer (15). A recent study reported that serum Cu level in BC patients was significantly higher than in healthy controls and patients with benign breast diseases (16), and copper can be transported to lysyl oxidase (LOX) family members, thus contributing to cancer metastatic (17). Too little copper can injure the function of important copper-binding enzymes, and copper accumulation can overwhelm a cell, leading to death (18). Recently, this novel mode of cell death named cuproptosis draws much attention, which is depicted that copper can bind to the lipoylated components of the tricarboxylic acid (TCA) cycle, leading to toxic protein stress and finally to cell death (19). Cuproptosis is different from other known death forms, including apoptosis, ferroptosis, and necroptosis, which is mediated by an ancient mechanism named protein lipoylation instead of adenosine triphosphate production. Furthermore, based on a whole-genome CRISPR-Cas9 technical screening, seven genes (FDX1, DLD, DLAT, LIAS, LIPT1, PDHA1, and PDHB) were found to be resistant to cuproptosis, while three genes (MTF1, GLS, and CDKN2A) sensitized the cells to cuproptosis (19).Among them, LIPT1 was found to be positively related to PD-L1 expression and negatively correlated with Treg cell infiltration in melanoma (20), whereas FDX1 expression was closely associated with six types (including T cells, monocytes, macrophages, mast cells) in renal cancer and five types (including CD8+ T cells, regulatory T cells, dendritic cells, mast cells) in thyroid carcinoma (THCA) (21, 22). However, the relationship between cuproptosis-related genes (CRGs) and immune infiltration of breast cancer remains unclear.

In our current study, we comprehensively analyzed the expression profile and stage characteristics of these CRGs in breast cancer, finding that CDKN2A, PDHA1 and LIPT1 expression were associated with pathological stage of BC. Then, we evaluated the prognostic value of CRGs by Kaplan-Meier Plotter and Cox analyses, finding that upregulated PDHA1 was associated with lower overall survival (OS) and recurrence-free survival (RFS) in BC. Moreover, we also conducted the correlation analysis between CRGs expression and immune cell infiltration, and the result showed that PDHA1 expression was strongly linked to CD4+ memory T cell, macrophage M0 and M1 cell, and mast cell in BC. These findings demonstrated that PDHA1 is a promising prognostic biomarker and actively takes part in the process of the immune response of breast cancer, thereby comprehensively shedding light on the exploitation of specific target drugs and immunegenic-mediated network of BC.

## Materials and methods

### Gene and protein expression profile

We obtained CRGs gene expression data and clinical information in all types of tumors and paired normal samples from The Cancer Genome Atlas (TCGA) and the Genotype-Tissue (GTEx). Meanwhile, comparison of CRG expression between 113 normal and 1109 breast cancer patients was also explored using Wilcoxon rank sum test. Furthermore, gene Expression Profiling Interactive Analysis 2 (GEPIA2) tool was used to analyze the CRGs expression in different pathological stages of BC (23). The university of alabama at birmingham cancer data analysis portal (UALCAN) tool was utilized to perform protein expression analysis of CRGs extracted from the Clinical Proteomic Tumor Analysis Consortium (CPTAC) dataset, including 18 normal samples and 125 primary BC samples (24, 25).

### Survival prognosis analysis and cox regression analysis

We used Kaplan–Meier Plotter to explore the prognostic value of CRGs expression for OS and RFS in BC. The Cox proportional hazard model was used to evaluate whether the expression of CRGs was correlated with clinical prognosis of BC patients. Hazard ratios (HR) > 1 and p < 0.05 suggested a significant association between CRGs and increased risk of death.

### Genetic alteration analysis

The cBioPortal database has an abundant resource for exploring and analyzing multidimensional cancer genomics data including epigenetic, gene expression profile and proteomic data (26). Therefore, the cBioPortal was used to evaluate the alteration frequency and form of CRGs in 996 BC samples.

### Immune subtype and tumor microenvironment (TME) analysis

The “limma” “ggplot2” and “reshape2” R packages were used to conduct the immune subtype analysis of CRGs. The p value < 0.05 was considered to indicate a significant difference. Meanwhile, we obtained the immune score, stromal score, and estimate score of different tumor samples by using the “estimate” and “limma” R packages. Correlation analysis between CRGs expression and estimate score of 33 TCGA tumors including BC patients was performed. Furthermore, we combined gene expression data with stemness score of RNAss and DNAss to conduct Spearman correlation test. Finally, the association between 10 CRGs and RNAss/DNAss of 33 TCGA tumors were obtained.

### Correlation analysis of the tumor-infiltrating immune cells

CIBERSORT algorithm was utilized to analyze 22 kinds of tumor-infiltrating immune cells (TIICs) in 33 TCGA tumors, such as regulatory T cells, gammadelta T cells, macrophages, CD8+ T cells, naive CD4+ T cells, follicular helper T cells. Then we used Timer 2.0 database to estimate the correlation between specific immune cell infiltration and CRGs expression levels in BC. Correlation values and p values were calculated by purity-adjusted Spearman’s rank correlation test. These results were displayed as a heatmap and scatter plots.

### Correlation and enrichment analysis of CRGs

GeneMANIA is a flexible and powerful website which can explore gene function, and search interacted genes (27, 28). We utilized the GeneMANIA website to analyze and classify the interactions between CRGs and their correlate genes. WebGestalt is an online tool concentrating on enrichment analysis, which has various of enrichment analysis algorithms and supports an abundant database of functional annotations (29). In this study, we used the WebGestalt database to perform GO and KEGG enrichment analysis of CRGs.

### Cell culture

Human breast cancer cell lines MCF-7, BT-474 and BT-549 and normal human breast epithelial cell line MCF-10A were obtained from American Type Cultural Collection (ATCC). MCF-10A were maintained in DMEM/F12 (Solarbio Science & Technology Co., Ltd, Beijing, China) supplemented with 10% fetal bovine serum (FBS) and 1% penicillin and streptomycin solution plus 3.5 μg/mL human insulin, 20 ng/mL epidermal growth factor and 0.5 μg/mL hydrocortisone. MCF-7, BT-474 and BT-549 were maintained in RPMI 1640 (Solarbio Science & Technology Co., Ltd, Beijing, China) supplemented with 10% fetal bovine serum (FBS) and 1% penicillin and streptomycin solution. These cells were cultured in an incubator with a 5% CO2 humidified atmosphere at 37°C. All reagents were commercially obtained from the Procell Life Science&Technology Co., Ltd (China)

### Quantitative real-time polymerase chain reaction analysis

Total RNA was extracted from the cells using TRIzol reagent (Thermo Fisher, USA). Reverse transcription kit and SYBR qPCR Master Mix (Shandong Sparkjade Biotechnology Co., Ltd.) were used for the cDNA synthesis of the target genes according to the manufacturer’s instructions. RT-qPCR primer sequences are listed in **Table S1**. Beta-actin gene expression was used as the endogenous control. The relative expression of the target genes relative to the control was calculated according to the 2^-ΔΔCT^ formula. Each experiment was conducted in triplicate.

### Tissue microarray and immunohistochemistry

The tissue microarray including 30 BC tissues (Outdo Biobank, Shanghai, HBre-Duc060CS-04) was used in the study. The tissue microarray samples were immunostained by PDHA1 antibody (Abclonal, Cat.A1895, dilution 1:250). All immunostained slides were scanned on AxioScan Z1 (Zeiss), and computerized image analysis was performed by Aipathwell. The degree of immunostaining was analyzed and scored by two independent pathologists who were blinded to the clinical details.

## Result

### Gene expression analysis data of CRGs

**Figure 1:**
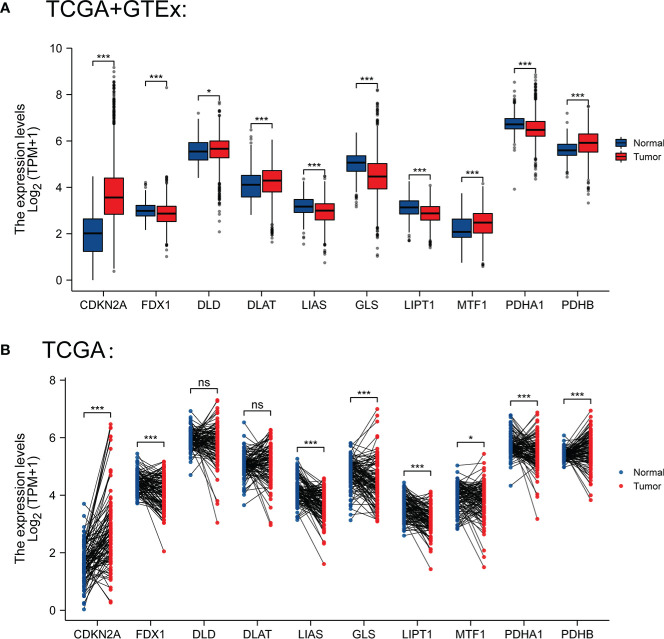
**(A)** Expression levels of CRGs in BC and normal tissues from TCGA and GTEx database (tumor in red and normal in blue) **(B)** Expression levels of CRGs in BC and paired normal tissues from TCGA. *P < 0.05 and ***P < 0.001; ns, No statistical significant.

**Figure 2:**
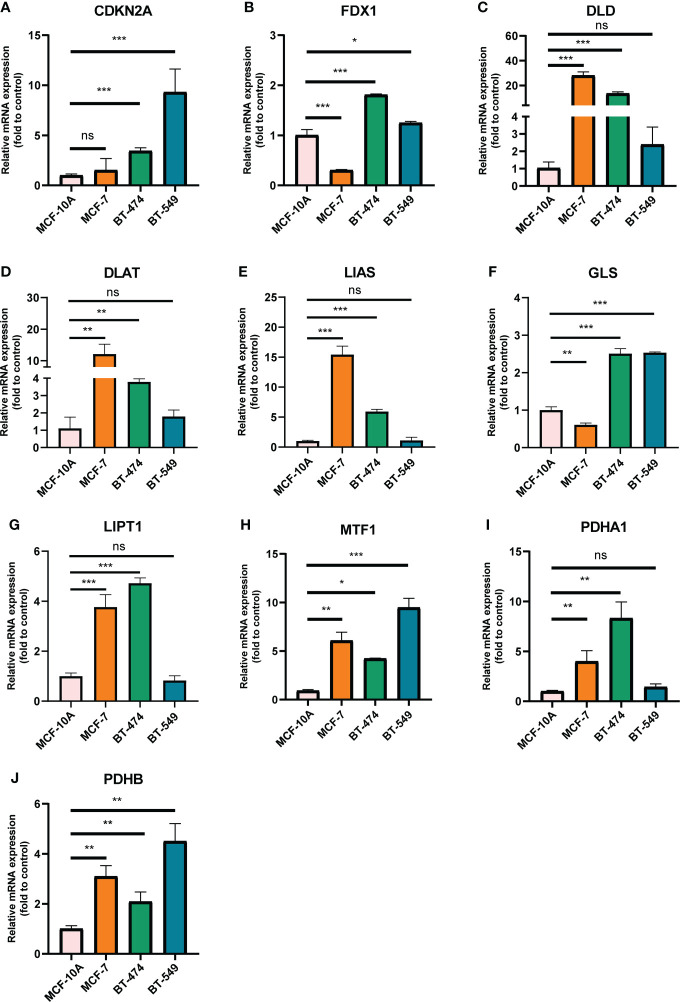
mRNA levels of CRGs in BC cell lines and human normal mammary epithelial cell quantified by real-time PCR. **(A)** CDKN2A; **(B)** FDX1; **(C)** DLD; **(D)** DLAT; **(E)** LIAS; **(F)** GLS; **(G)** LIPT1; **(H)** MTF1; **(I)** PDHA1; **(J)** PDHB. The experiments were repeated three times. *p < 0.05, **p < 0.01, ***p < 0.001; ns, no statistically significant.

We selected 10 genes (CDKN2A, DLAT, FDX1, DLD, LIPT1, LIAS, GLS, PDHB, MTF1 and PDHA1) which are closely related to cuproptosis and next performed expression analysis in breast cancer (19). As displayed in **Figure 1A**, we analyzed the expression pattern of CRGs in breast cancer tissues and non-tumor tissues based on TCGA and GTEx dataset, illustrating that CDKN2A, DLD, DLAT, MTF1 and PDHB expression were significantly elevated in breast cancer compared with their normal tissues. However, FDX1, LIAS, GLS, LIPT1 and PDHA1 were highly expressed in breast normal tissues (P < 0.05). Additionally, the diversity of the tumor tissues and adjacent normal tissues from TCGA database was also examined in **Figure 1B**, the expression discrepancy of DLD and DLAT has no statistical significance and others are consistent with the above results. Subsequently, normal breast epithelial cell line (MCF-10A) and three breast cancer cells with different receptor expressed (ER+ BC cell line MCF-7, HER2+ BC cell line BT474 and triple-negative breast cancer (TNBC) cell line BT549) were chosen to detect cuproptosis-related gene expression *via* RT-qPCR experiments, suggesting that the expression levels of MTF1 and PDHB were significantly higher in three above kinds of BC cells than in MCF-10A, whereas DLD and DLAT were higher expressed in MCF-7 and BT-474 than in MCF-10A. Notedly, there was no difference in LIPT1/PDHA1 expression between MCF-10A and BT-549 cells, but their expression in receptor-positive breast cancer cells was higher than that of MCF-10A, which might indicate that the difference of LIPT1 and PDHA1 expression was related to the receptor status of BC (**Figure 2**).

**Figure 3:**
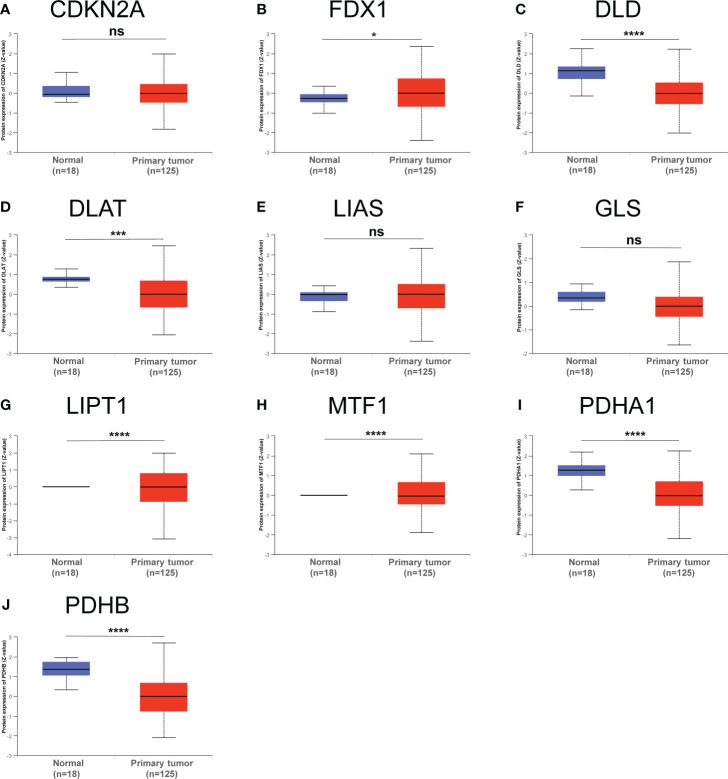
Total protein levels of CRGs **(A–J)** in normal tissue and BC. Protein expresssion data was collected and analyzed using CPTAC. *p < 0.05, ***P < 0.001, ****P < 0.0001; ns, no statistically significant.

**Figure 4:**
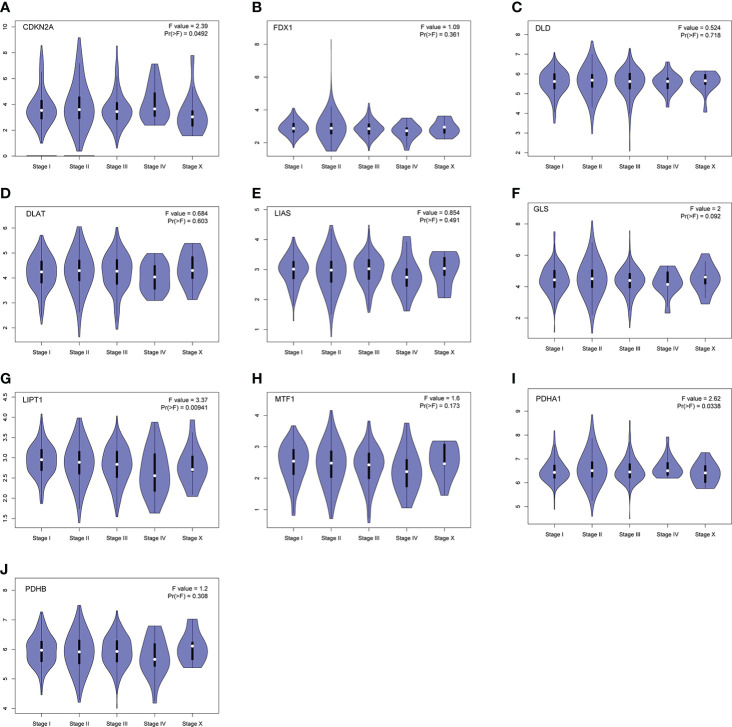
Stage-dependent expression levels of CRGs **(A–J)**. Main pathological stages of BC were assessed and compared using TCGA data. The log2 (TPM + 1) for log-scale was used.

Furthermore, we also obtained CRGs expression data in 18 types of cancers (BLCA, BRCA, CHOL, COAD, ESCA, LUAD, GBM, HNSC, KIRC, UCEC, READ, KICH, LIHC, KIRP, LUSC, THCA, PRAD, and STAD) from TCGA dataset, showing that CRGs had a rich heterogeneity in these cancers. As displayed in **Figure S1**, CDKN2A was highly expressed in most cancers. In contrast, FDX1 and MTF1 expression in most cancers were lower than paired normal tissues. In addition to transcription, we also analyzed protein levels of CRGs using the large-scale proteome data available based on CPTAC dataset. The result demonstrated that the total protein expression levels of FDX1, LIPT1 and MTF1 in BC were significantly higher than the corresponding control tissues. Nonetheless, DLD, DLAT, PDHA1 and PDHB protein expression in BC tissues were lower than normal breast tissues (**Figure 3**). These results illustrate that expression differences of FDX1, LIPT1, MTF1, PDHA1 and PDHB genes may be involved in the development and outcome of BC. Additionally, we also analyzed the association between CRGs expression and pathological stage of BC patients by GEPIA2 tool, displaying stage-specific expressional changes of CDKN2A, LIPT1 and PDHA1 in BC patients (**Figure 4**). Then, early-stage associated prognosis analysis of the above three genes was performed in this study and found that only PDHA1 expression was closely related to survival rate of these patients (**Figure S2**). Previous studies demonstrated that PDHA1 could regulate the growth of breast cancer cells by the coordination of glucose metabolism reprogramming (30).To further elucidate the role of PDHA1 expression in the clinical features of BC patients, we verified that PDHA1 expression was associated with T stage from the database and subsequently collected 30 breast cancer samples to confirm that PDHA1 expression was higher in T2 stage than in T1 stage (**Figure S3**). In conclusion, PDHA1 is expected to be a potential molecule for early pathological diagnosis of BC.

### CRGs prognostic value in BC

**Figure 5:**
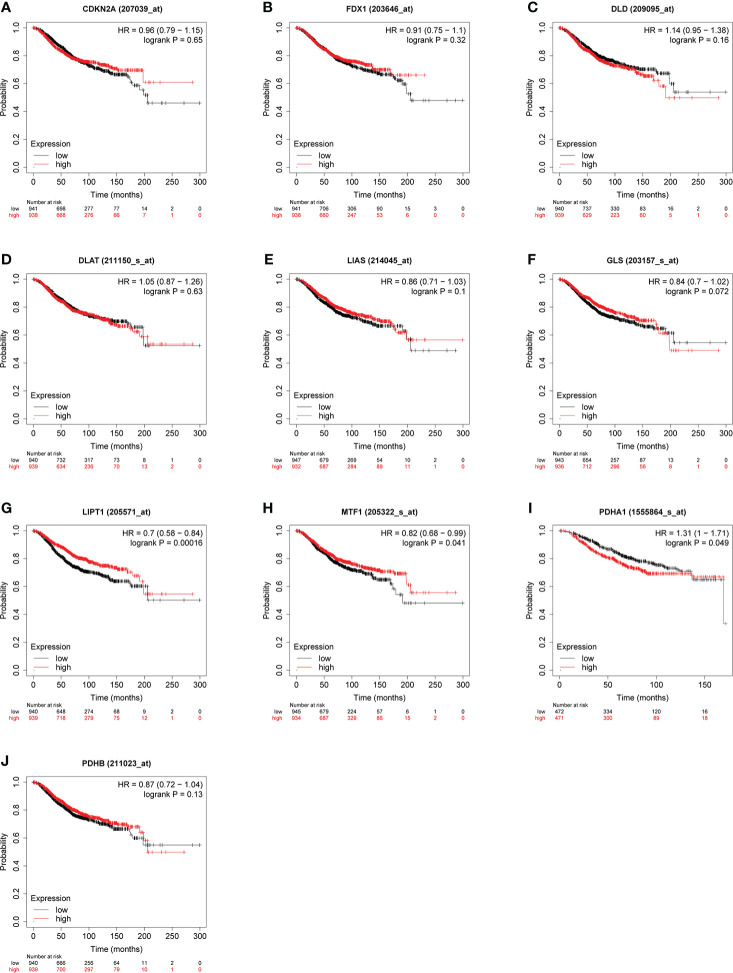
Relationship between CRGs **(A–J)** expression levels and OS in BC. The curves generated by using the KM plotter database show the prognostic value of CRGs. The red lines indicate high CRGs expression, and the black lines indicate low CRGs expression.

**Figure 6:**
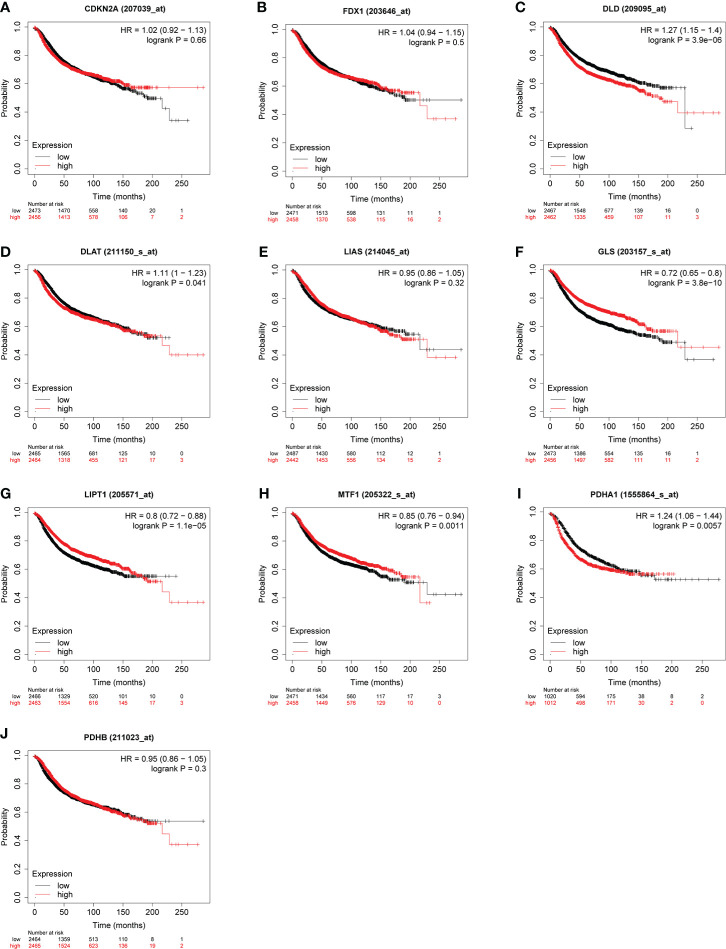
Correlation between CRGs **(A–J)** expression levels and RFS in BC. The curves generated by using the KM plotter database show the prognostic value of CRGs.

Some evidence suggested PDHA1 was closely related to the progression and prognosis of gastric, pancreatic and esophageal squamous cancer (31–33). Therefore, we performed Kaplan-Meier analysis between CRGs expression and survival outcomes in BC, including OS and RFS. The results of OS analysis suggested that high expression of PDHA1 was correlated with the worse endpoint of BC patients, but upregulation of LIPT1 and MTF1 was associated with the better endpoint in BC (**Figure 5**). We also verified that the expression of these three genes was actively involved in the relapse free survival of BC (**Figure 6**)

To further clarify the effect of LIPT1 and PDHA1 on the prognosis of breast cancer, we performed univariate and multivariate Cox analysis of OS in BC patients, and the results are shown in **Table 1**. In univariate Cox model, T stage (P = 0.012), N stage (P < 0.001), M stage (P < 0.001) and PDHA1 expression (P = 0.025) were correlated with OS in BC patients. The result of multivariate Cox analysis displayed that N stage (P = 0.011), M stage (P = 0.049) and PDHA1 expression (P = 0.014) were still associated with worse clinical outcome. Therefore, PDHA1 is more possible to be seen as an independent prognostic biomarker in BC patients.

**Table 1:** Univariate and multivariate Cox analyses of prognostic factors in breast cancer.

Characteristics	Univariate analysis		Multivariate analysis	
	Hazard ratio (95% CI)	p value	Hazard ratio (95% CI)	p value
Age (<=60 vs >60)	2.020 (1.465-2.784)	**<0.001**	2.077 (1.440-2.997)	**<0.001**
Pathologic stage (I+ II vs III+ IV)	2.391 (1.703-3.355)	**<0.001**	1.913 (1.112-3.291)	**0.019**
T stage (T1+T2 vs T3+T4)	1.608 (1.110-2.329)	**0.012**	0.853 (0.507-1.437)	0.551
N stage (N0 vs N1+N2+N3)	2.239 (1.567-3.199)	**<0.001**	1.776 (1.143-2.760)	**0.011**
M stage (M0 vs M1)	4.254 (2.468-7.334)	**<0.001**	2.024 (1.004-4.082)	**0.049**
LIPT1: High vs Low	0.839 (0.609-1.155)	0.282		
PDHA1: High vs Low	1.444 (1.046-1.993)	**0.025**	1.575 (1.097-2.261)	**0.014**

The value in bold indicate that p is less than 0.05, which is meaningful.

### Genetic alteration analysis of CRGs in breast cancer

**Figure 7:**
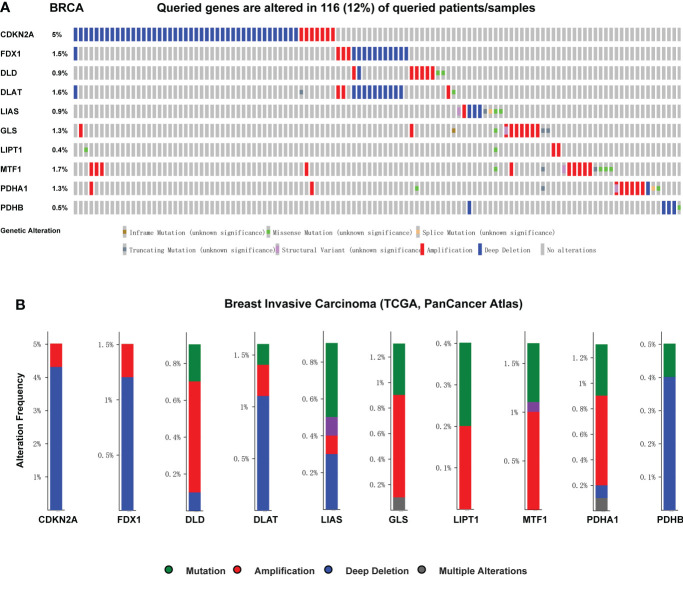
Genetic mutation and correlation analysis of CRGs in BC. **(A)** Profile of alteration rates for CRGs in BC using cBioPortal **(B)** Genetic alteration frequency data of CRGs in BC using cBioPortal.

We used cBioPortal database to perform the frequency and types of gene changes of CRGs in 996 BC samples. As displayed in **Figure 7A**, the highest variation rate of CRGs was CDKN2A, which is 5%. The genetic alteration rate of the LIPT1 gene was 0.4%, which was the lowest in the CRGs. In 996 BC samples, 116 patients had genetic alteration in CRGs, with a total variation rate of 15.1%. Gene mutation, deep deletion and amplification were the main genetic variation types in CRGs. The mainly alteration types of DLD, GLS, LIPT1, MTF1 and PDHA1 are gene amplification (**Figure 7B**).

### Immune subtype and tumor microenvironment (TME) analysis of CRGs

**Figure 8:**
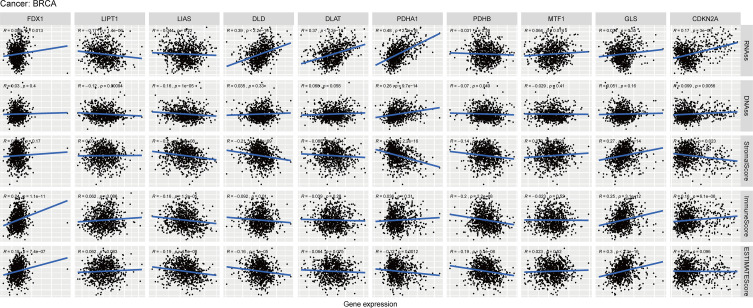
Relationship of CRGs expression with RNAss, DNAss, StromalScore, ImmuneScore and EstimateScore in BC, R represents correlation value, positive number represents positive correlation, negative number represents negative correlation.

As we know from **Figure 8**, the expression of FDX1, LIAS and GLS were positively correlated with estimate score, while DLD, PDHA1, PDHB were negatively associated with estimate score in BC. In those genes, PDHA1 was the most significantly correlated with immune score, and GLS was the most significantly correlated with stromal score.

PDHA1 and CDKN2A were positively correlated with RNAss and DNAss, while LIPT1 was negatively associated with RNAss and DNAss in BC. Furthermore, PDHA1 was the most importantly correlated with RNAss and DNAss. Then we conducted these analyses of CRGs in pan-cancer. The result showed that most of CRGs were positively correlated with the RNAss and DNAss in pan-cancer (**Figures S4A, B**). In addition, **Figures S4D–F** demonstrated that most of CRGs were significantly negatively associated with stromal score, immune score and estimate score in pan-cancer.

**Figure 9:**
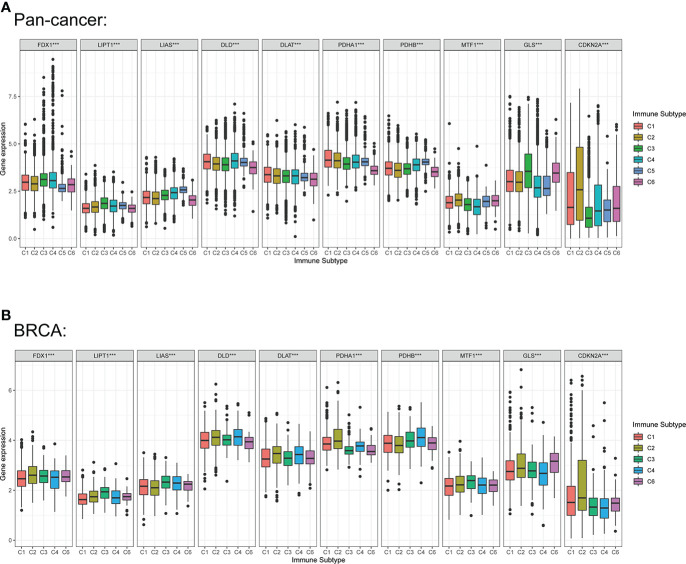
Correlation among expression levels of CRGs and different immune subtype in pan-cancer and BC. **(A)** CRGs expression levels in different immune subtypes in pan-cancer. **(B)** CRGs expression levels in different immune subtypes in BC. X axis is immune subtype, Y axis is gene expression. C1, wound healing; C2, IFN-γ dominant; C3, inflammatory; C4, lymphocyte depleted; C5, immunologically quiet; C6, TGF-β dominant. P < 0.05, ***P < 0.001.

Previous studies demonstrated that CRGs could regulate immune process in some specific tumors, including melanoma, liver cancer and renal cancer. Therefore, we compared the relationships between CRGs expression and immune subtype in this study. Immune subtype were classified into six types, including C1 (wound healing), C2 (IFN-gamma dominant), C3 (inflammatory), C4 (lymphocyte depleted), C5 (immunologically quiet) and C6 (TGF-β dominant) (34). The results suggested that expression of CRGs in pan-cancer were significantly different in these immune subtypes (**Figure 9A**). For BC, the overall expression of DLD, DLAT, PDHA1 and PDHB were the most obvious in the five subtypes. Notably, PDHA1 was the highest expressed in C2, and was the lowest in C6 (**Figure 9B**).

### Association between CRGs expression and immune cell infiltration

**Figure 10:**
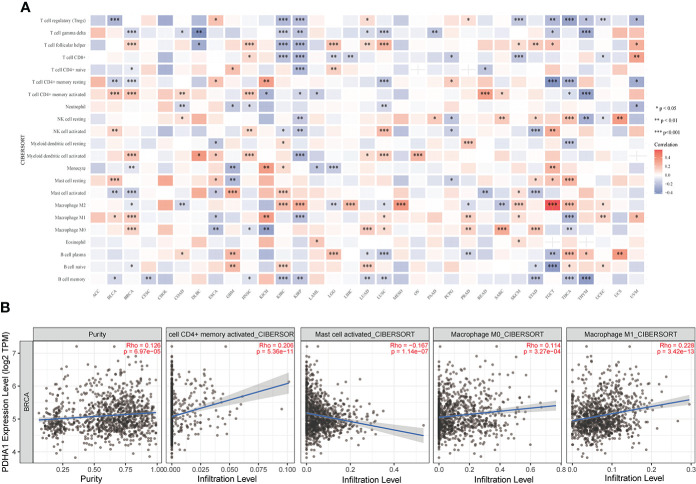
Correlation analysis of PDHA1 expression with immune cell infiltration analysis in BC. **(A)** The relationship between PDHA1 expression level and the infiltration levels of immune-related cells. **(B)** The scatter plots of relationship between PDHA1 expression and infiltration levels of immune-related cells by using TIMER2 database *P < 0.05, **P < 0.01, and ***P < 0.001.

As the crucial components of the TME, tumor-infiltrating immune cells have been found to be closely associated with the initiation, progression and metastasis of cancers (35, 36). To further assess the relationship between LIPT1 and PDHA1 and immune cell infiltration levels in pan-cancer, we used “CIBERSORT” algorithm to evaluate the 22 immune cells based on published data and found that the expression of PDHA1 was negatively associated with gamma delta T cell and memory B cell in most cancers (**Figure 10A**). Whereas LIPT1 expression was negatively correlated with regular T cell and macrophage M0 cell in most cancers (**Figure S5A**). Furthermore, we also used the TIMER2 database to verify the relationship between PDHA1 expression and immune cells in BC **(** **Figures 10B**), suggesting that the expression level of PDHA1 was positively associated with CD4+ memory T cell and macrophage cell, and negatively linked to mast cell active. However, LIPT1 expression has no significant difference in CD4+ memory T cell and macrophage M1 cell (**Figures S5B–E**). **Figure S6** displayed the correlation between other eight genes expression and immune cell infiltration. Additionally, correlation analysis between LIPT1/PDHA1 and related markers of immune cells of BC was also illustrated in this study, founding that PDHA1 has the most negative correlation with GATA3 and positive relation with CCR8 (**Table S2**). Nevertheless, the relationship of CRGs with significant immune checkpoint members including PD-1, PD-L1, PD-L2, LAG3 and CTLA4 in BC was also showed and found that PDHA1 has little correlation with these genes, which may suggest that PDHA1 regulates immune infiltration from other directions (**Figure S7**).

### Correlation and enrichment analysis of CRGs

**Figure 11:**
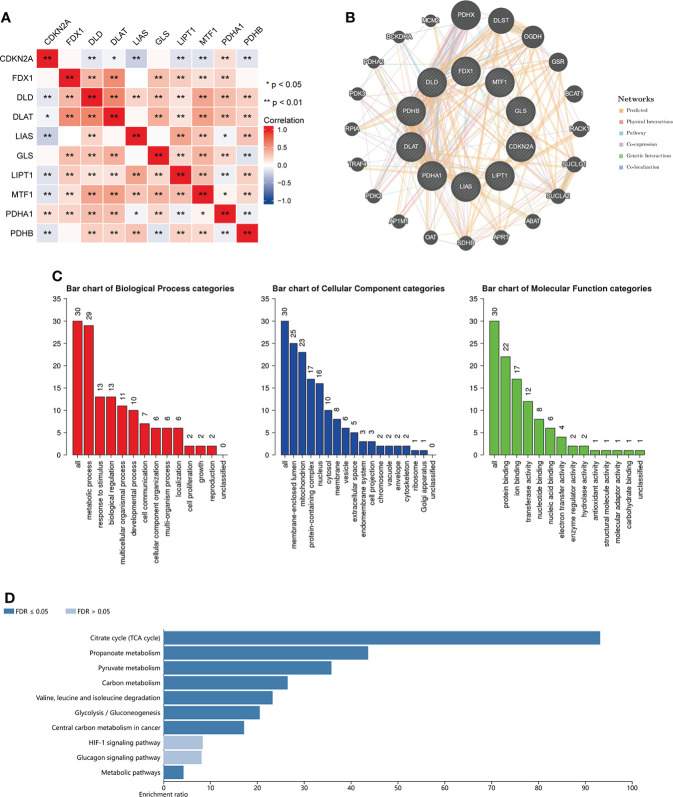
Enrichment analysis of functions and pathways of the related molecules of CRGs. **(A)** The relationship between the CRGs. Blue color indicates negative correlation and red color indicate positive correlation. **(B)** protein–protein interaction network of the CRGs analyzed by using GeneMANIA; **(C)** GO analysis of relevant biological processes, cellular components, and molecular functions of the CRGs; **(D)** KEGG pathway analysis of the relevant signal pathways *P < 0.05, **P < 0.01.

The CRGs correlation analysis in BC indicated that CDKN2A expression was negatively associated with the expression of other six CRGs (DLD, DLAT, LIAS, LIPT1, MTF1 and PDHB). The other nine genes expression except for CDKN2A were positively correlated with the expression of most CRGs (**Figure 11A**). Furthermore, we conducted the correlation analysis in pan-cancer, and the result demonstrated that DLD and PDHA1 were the two genes having the most significantly positive association (Correlation coefficient = 0.39). CDKN2A and GLS, CDKN2A and LIAS, PDHA1 and MTF1 were the genes having the most significantly negative correlation (Correlation coefficient= -0.17, **Figure S8C**). **Figure S8A** displayed the expression heatmap of the ten genes in 18 TCGA pan-cancers. PDHA1 was the highest expression in LUSC, and LIPT1 was the highest expression in GBM. **Figure S8B** demonstrated that PDHA1 was the highest expression in pan-cancer, and CDKN2A was the lowest expression in pan-cancer.

Then we used the GeneMANIA database to find the potential interaction partners with CRGs. The result showed that expression of CRGs was closely associated with PDHX, DLST, OGDH and so on (**Figure 11B**). We also used the WebGestalt database to perform GO and KEGG functional enrichment analysis of the above 20 genes associated with CRGs. GO analysis displayed that metabolic process, response to stimulus and biological regulation were involved in biological processes. The result of KEGG analysis showed that Citrate cycle, Propanoate metabolism and Pyruvate metabolism were revealed as the three most importantly enriched pathways, suggesting that these genes were critically involved in the cell metabolic process (**Figures 11C, D**).

## Discussion

Cuproptosis occurs *via* directly binding of copper to lipoylated components of the tricarboxylic acid cycle (19). The procedure of cuproptosis is significantly associated with mitochondrial respiration. Abundant copper within cells is transported to the mitochondria by ionophores and combined with lipoylated components of the tricarboxylic acid cycle, leading to the accumulation of lipoylated proteins, thereby resulting in cell death because of proteotoxic stress. Preliminary studies on the function of CRGs have been explored in several tumors including bladder cancer (37), esophageal carcinoma (38), melanoma (20) and liver cancer (39). However, the role of CRGs in the development of TME and their potential prognostic value in BC remains unknown.

Our study firstly conducted expression patterns and clinical characteristic analysis of CRGs and found that CDKN2A, LIPT1 and PDHA1 were associated with the stages of BC. Then, survival analysis was performed for the evaluation of prognostic role of the CRGs, and the result showed that the expression of PDHA1, LIPT1 and MTF1 were associated with the OS and RFS in BC, however, the COX analysis and T-stage associated survival analysis suggested only PDHA1 expression was significantly related to the prognosis of BC patients. Besides, previous studies have suggested that the expression of CRGs was closely associated with TME in various malignancies (37, 40), and our study also found that FDX1, DLAT, PDHA1, GLS, CDKN2A were the highest expressed in C2, and LIPT1, LIAS, MTF1 were the highest expressed in C3. LIAS, DLD, DLAT and PDHB were negatively related to stromal score, immune score and estimate score, and FDX1, LIPT1 and GLS were positively related to three scores. These results demonstrating that CRGs were closely correlated with TME in BC.

Regulatory T cell (Tregs), which can express Foxp3, plays a pivotal role in maintaining of self-tolerance by restraining immune responses to self or xenogenous antigens (41–43). Tumor-infiltrating Tregs can contribute to poor clinical endpoint, which result in progression of tumor through inhibiting antitumor immunity and promoting angiogenesis (44). Hence, they are considered as a major obstacle to the successful application of cancer immunotherapy (45). In our study, FDX1, GLS, DLAT, MTF1 and LIPT1 were positively associated with main immune checkpoint genes expression and negatively correlated with the infiltration level of Tregs, which suggesting that they could be a positive biomarker for the prognosis of BC patients treated by immunotherapy.

Pyruvate dehydrogenase (PDH) plays a crucial role in glucose metabolism by oxidatively decarboxylating pyruvate to produce acetyl-CoA for the TCA cycle. As the catalytic component of pyruvate dehydrogenase (PDH), pyruvate dehydrogenase E1 (PDHA1) is located in the X chromosome and loss-of-function PDHA1 mutation appear serious lactic acidosis (46, 47). Besides, PDHA1 serves as a significant bridge between glycolysis and the mitochondrial TCA cycle. Emerging proofs have illustrated that PDHA1 dysregulation can promote glucose metabolism reprogramming and remodel cellular metabolic pattern (30, 48–50). In terms of its effect on cancer progression and development, decreased expression of PDHA1 was verified to be associated with an unfavorable outcome in a variety of types of cancers including ovarian (51), liver (52) and esophageal cancer (53). Furthermore, knockout of PDHA1 led to greater Warburg effect and more malignant characteristic on esophageal squamous cancer (50). In our study, we found that mRNA and protein expression level of PDHA1 was lower in BC tissues than in normal breast tissues. Kaplan-Meier analysis and Cox analyses of PDHA1 demonstrated that upregulated expression of PDHA1 was associated with worse OS and RFS outcome of BC patients, illustrating that PDHA1 has the potential to serve as a prognosis biomarker of BC.

We also analyzed the potential association between PDHA1 gene expression and immune infiltration in 33 types of cancers. In our study, we found that PDHA1 expression was negatively associated with the infiltration of regulate T cell (Tregs) in 9 tumors. Therefore, increased PDHA1 might promote the response to immunotherapy by inhibiting regulate T cell infiltration in the tumor microenvironment. The population of tumor-associated macrophages (TAMs) are the most abundant among tumor-infiltrating immune cells in TME and generally polarize into two different function subtypes termed as classically activated M1 and alternatively activated M2 subtypes (54). The M1-like macrophages, show antitumor characteristic by secreting pro-inflammatory cytokines (such as TNF and interleukin-2) and reactive nitrogen and oxygen intermediates (55). On the contrary, the M2-like macrophages are activated by the type 2 T helper cell (Th2) cytokines such as IL-4, IL-10 and IL-13, and exhibit promoting tumor capacity. In this study, we found that PDHA1 expression was positively correlated with macrophage M0 and M1 cell *via* using TIMER2 database, and negatively linked to macrophage M2 cell in BC. Based on these data, we considered that PDHA1 may have direct or indirect effects on macrophage polarization, thereby regulating the biological process and clinical outcome of BC, which is need to be confirmed by more robust and sufficient molecular experiments.

In conclusion, we conducted the expression profile analysis and prognosis analysis of the CRGs in BC, finding that PDHA1 expression was downregulated in BC. The higher expression of PDHA1 was associated with worse clinical endpoint of BC patients. Furthermore, the immune infiltration analysis demonstrated that PDHA1 was closely associated with the CD4+ memory T cell, macrophage M0 and M1 cell, and mast cell in BC. These results may provide insights for further investigation of the PDHA1 as potential target in BC.

## Data availability statement

The original contributions presented in the study are included in the article/**Supplementary Material**. Further inquiries can be directed to the corresponding authors.

## Author contributions

ZhiS and ZY designed the overall study and revised the paper. TH, YL and JL drafted the manuscript and performed the data analysis. BS and ZheS participated in the data collection. All authors contributed to the article and approved the submitted version.

## Funding

This project is supported by the grand “National Natural Science Foundation of China.” No. 81803044.

## Acknowledgments

We thank the supporting of the grants from the National Natural Science Foundation of China (No. 81803044) and School of Medicine, Xiamen University, Xiamen, China.

## Conflict of interest

The authors declare that the research was conducted in the absence of any commercial or financial relationships that could be construed as a potential conflict of interest.

## Publisher’s note

All claims expressed in this article are solely those of the authors and do not necessarily represent those of their affiliated organizations, or those of the publisher, the editors and the reviewers. Any product that may be evaluated in this article, or claim that may be made by its manufacturer, is not guaranteed or endorsed by the publisher.
