# Supplementary material for: Insights into prognosis and immune infiltration of cuproptosis-related genes in breast cancer
Source: Front Immunol. 2022 Nov 28;13:1054305. doi: 10.3389/fimmu.2022.1054305 (PMC9742524; doi:10.3389/fimmu.2022.1054305)
Supplement: Supplementary file 1 [file DataSheet_1.docx]

**Supplementary Material**

**Supplementary Figures**

**Table S1:**

Quantitative Real-Time PCR primers used in the study

| **Name** | **Sequences of Primer** |
| --- | --- |
| CDKN2A Forward Primer | GATCCAGGTGGGTAGAAGGTC |
| CDKN2A Reverse Primer | CCCCTGCAAACTTCGTCCT |
| FDX1 Forward Primer | TTCAACCTGTCACCTCATCTTTG |
| FDX1 Reverse Primer | TGCCAGATCGAGCATGTCATT |
| DLD Forward Primer | CTCATGGCCTACAGGGACTTT |
| DLD Reverse Primer | GCATGTTCCACCAAGTGTTTCAT |
| DLAT Forward Primer | CGGAACTCCACGAGTGACC |
| DLAT Reverse Primer | CCCCGCCATACCCTGTAGT |
| LIAS Forward Primer | GTATGTGAGGAAGCTCGATGTC |
| LIAS Forward Primer | CACCCATCAACATGATCGTGG |
| GLS Forward Primer | AGGGTCTGTTACCTAGCTTGG |
| GLS Reverse Primer | ACGTTCGCAATCCTGTAGATTT |
| LIPT1 Forward Primer | TTGCTAAAGAGCCCTTACCAAG |
| LIPT1 Reverse Primer | TCATCCGTTGGGTTTATTAGGTG |
| MTF1 Forward Primer | CAGTGCGGAGAACACTTGC |
| MTF1 Reverse Primer | CAGTGCGGAGAACACTTGC |
| PDHA1 Forward Primer | TGGTAGCATCCCGTAATTTTGC |
| PDHA1 Reverse Primer | ATTCGGCGTACAGTCTGCATC |
| PDHB Forward Primer | AGTGGTGGTGCTAGAGAATGA |
| PDHB Reverse Primer | TGCAGCTTCTAAGCAGTGGC |

**Table S2:**

Correlation analysis between LIPT1/PDHA1 and relate genes and markers of immune cells in TIMER.

| **Description** | **Gene markers** | **LIPT1** |  |  |  | **PDHA1** |  |  |  |
| --- | --- | --- | --- | --- | --- | --- | --- | --- | --- |
| \|  \| \| --- \| |  | **None** |  | **Purity** |  | **None** |  | **Purity** |  |
|  |  | **Cor** | **P** | **Cor** | **P** | **Cor** | **P** | **Cor** | **P** |
| \| CD8+ T cell \| \| --- \| | CD8A  CD8B | 0.205  0.117 | ***  *** | 0.162  0.063 | ***  * | 0.02  0.07 | ns  * | 0.098  0.158 | **  *** |
| T cell (general) | CD3D  CD3E | 0.11  0.141 | ***  *** | 0.052  0.086 | ns  ** | 0.01  0.025 | ns  ns | 0.088  0.111 | **  *** |
|  | CD2 | 0.162 | *** | 0.113 | *** | 0.047 | ns | 0.131 | *** |
| B cell | CD19  CD79A | 0.065  0.095 | *  ** | 0.014  0.044 | ns  ns | 0.046  0.028 | ns  ns | 0.112  0.104 | ***  *** |
| Monocyte | CD86  CD115 (CSF1R) | 0.099  0.127 | **  *** | 0.056  0.078 | ns  * | 0.084  -0.063 | **  * | 0.142  -0.006 | ***  ns |
| TAM | CCL2  CD68 | 0.113  -0.015 | ***  ns | 0.088  -0.062 | **  * | 0.098  0.043 | **  ns | 0.151  0.088 | ***  ** |
|  | IL10 | 0.062 | * | 0.033 | ns | 0.115 | *** | 0.163 | *** |
| M1 Macrophage | INOS (NOS2)  IRF5 | -0.037  0.012 | ns  ns | -0.033  -0.018 | ns  ns | 0.067  0.093 | *  ** | 0.08  0.128 | *  *** |
|  | COX2 (PTGS2) | 0.158 | *** | 0.145 | *** | 0.023 | ns | 0.076 | * |
| M2 Macrophage | CD163  VSIG4 | -0.001  0.038 | ns  ns | -0.045  0.002 | ns  ns | 0.139  0.012 | ***  ns | 0.193  0.059 | ***  ns |
|  | MS4A4A | 0.086 | ** | 0.047 | ns | 0.067 | * | 0.131 | *** |
| Neutrophils | CD66b (CEACAM8) | 0.073 | * | 0.069 | * | 0.029 | ns | 0.041 | ns |
|  | CCR7 | 0.107 | *** | 0.057 | ns | 0.009 | ns | 0.079 | * |
|  | CD11b  (ITGAM) | 0.152 | *** | 0.12 | *** | -0.034 | ns | 0.009 | ns |
| Th1 | T-bet (TBX21)  STAT4 | 0.14  0.245 | ***  *** | 0.085  0.212 | **  *** | 0.048  0.026 | ns  ns | 0.133  0.11 | ***  *** |
|  | STAT1 | 0.188 | *** | 0.16 | *** | 0.187 | *** | 0.22 | *** |
|  | IFN-γ (IFNG) | 0.117 | *** | 0.078 | * | 0.137 | *** | 0.199 | *** |
|  | TNF-α (TNF) | 0.065 | * | 0.04 | ns | 0.169 | *** | 0.2 | *** |
| Th2 | GATA3 | 0.235 | *** | 0.268 | *** | -0.29 | *** | -0.339 | *** |
|  | STAT6 | 0.291 | *** | 0.288 | *** | -0.141 | *** | -0.119 | *** |
|  | STAT5A | 0.241 | *** | 0.203 | *** | -0.062 | ns | -0.018 | ns |
|  | IL-13 | 0.049 | ns | 0.041 | ns | 0.099 | *** | 0.117 | *** |
| Treg | FOXP3  TGFβ (TGFB1) | 0.058  0.09 | ns  ** | 0.021  0.045 | ns  ns | 0.187  -0.24 | ***  *** | 0.246  -0.21 | ***  *** |
|  | CCR8 | 0.087 | ** | 0.064 | * | 0.209 | *** | 0.25 | *** |
|  | STAT5B | 0.321 | *** | 0.307 | *** | -0.036 | ns | -0.013 | ns |
| T cell exhaustion | PD-1 (PDCD1) | 0.074 | * | 0.016 | ns | 0.061 | * | 0.13 | *** |
|  | CTLA4 | 0.064 | * | 0.016 | ns | 0.17 | *** | 0.24 | *** |
|  | LAG3 | -0.018 | ns | -0.056 | ns | 0.182 | *** | 0.219 | *** |
|  | TIM-3  (HAVCR2) | 0.085 | ** | 0.044 | ns | 0.041 | ns | 0.093 | ** |
|  | GZMB | 0.007 | ns | -0.052 | ns | 0.151 | *** | 0.215 | *** |

TAM, tumor-associated macrophage; Th, T helper cell; Treg, regulatory T cell; Cor, R value of Spearman's correlation; NS, correlation without adjustment; Purity, correlation adjusted by purity.

*P < 0.05

**P < 0.01

***P < 0.001

**FIGURE S1**


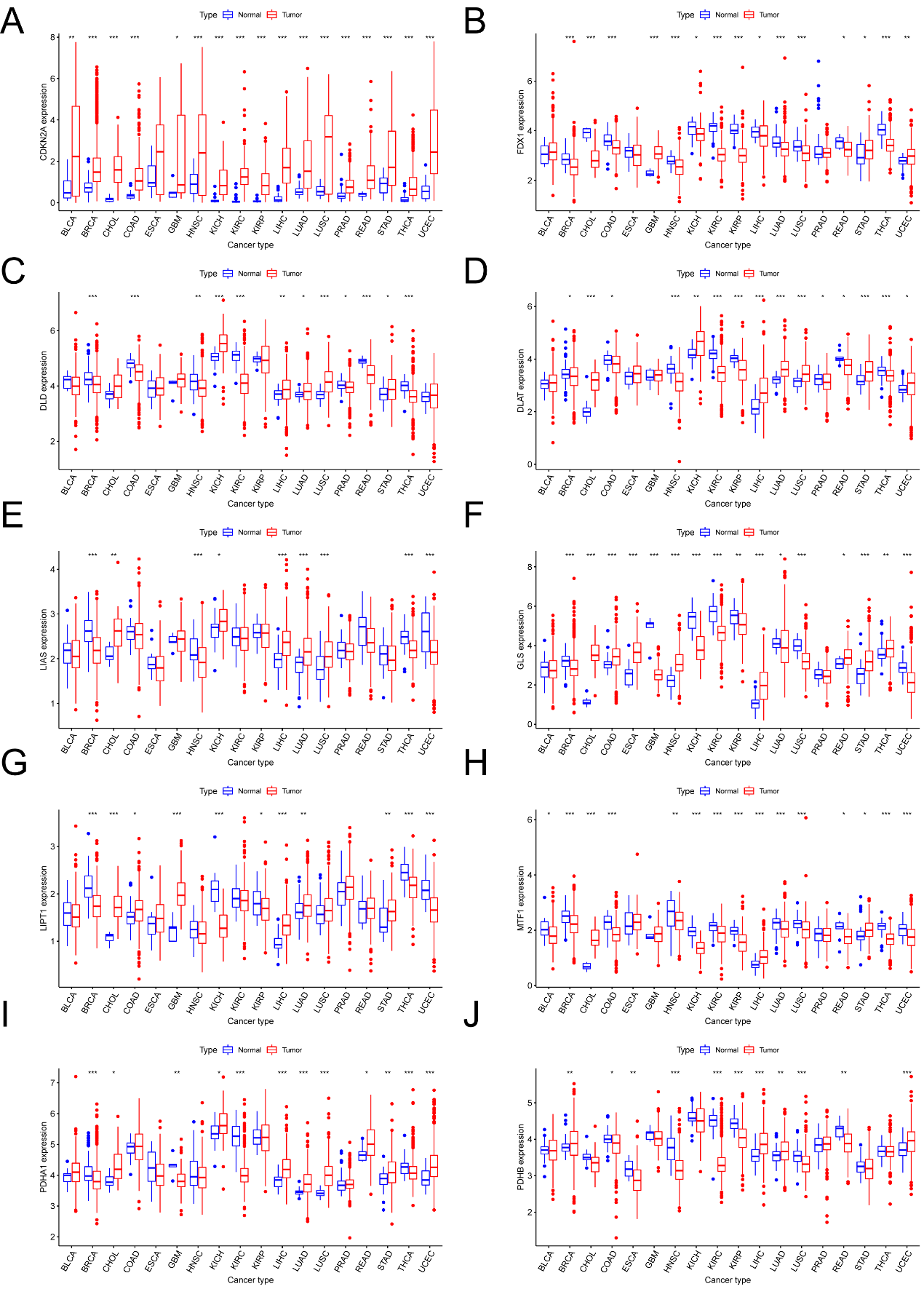


**FIGURE S2**

**
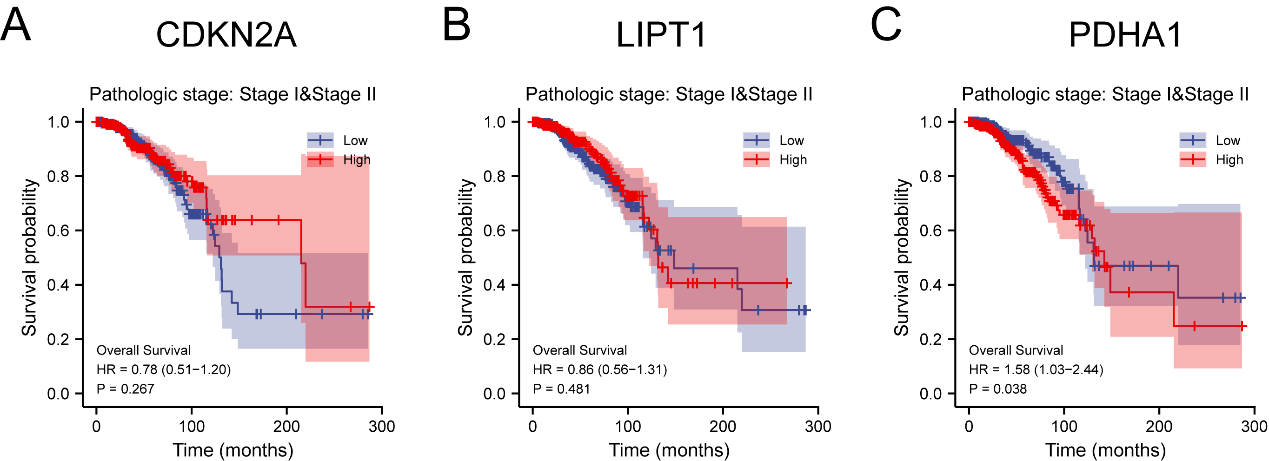
**

**FIGURE S3**

**
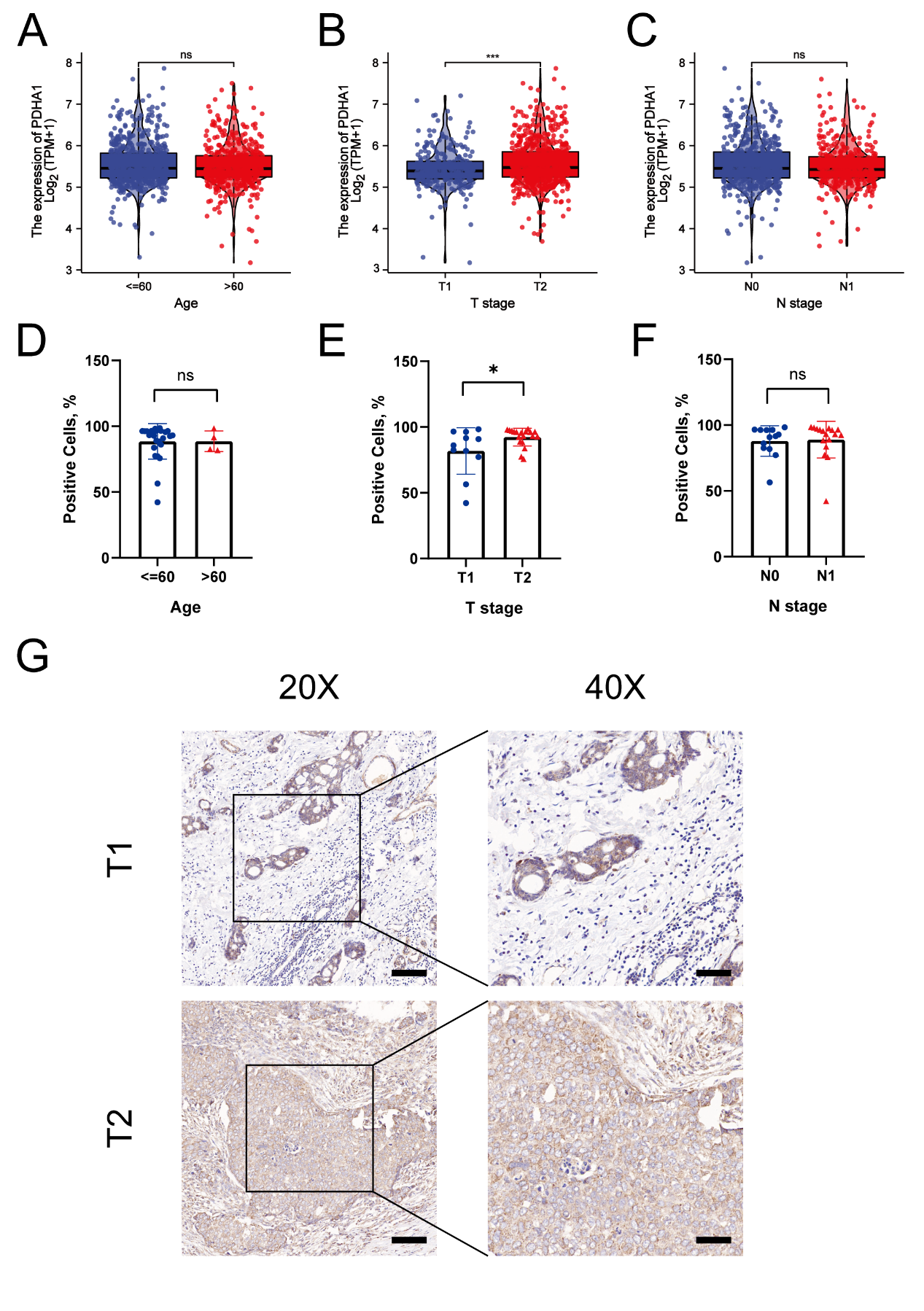
**

**FIGURE S4**

**
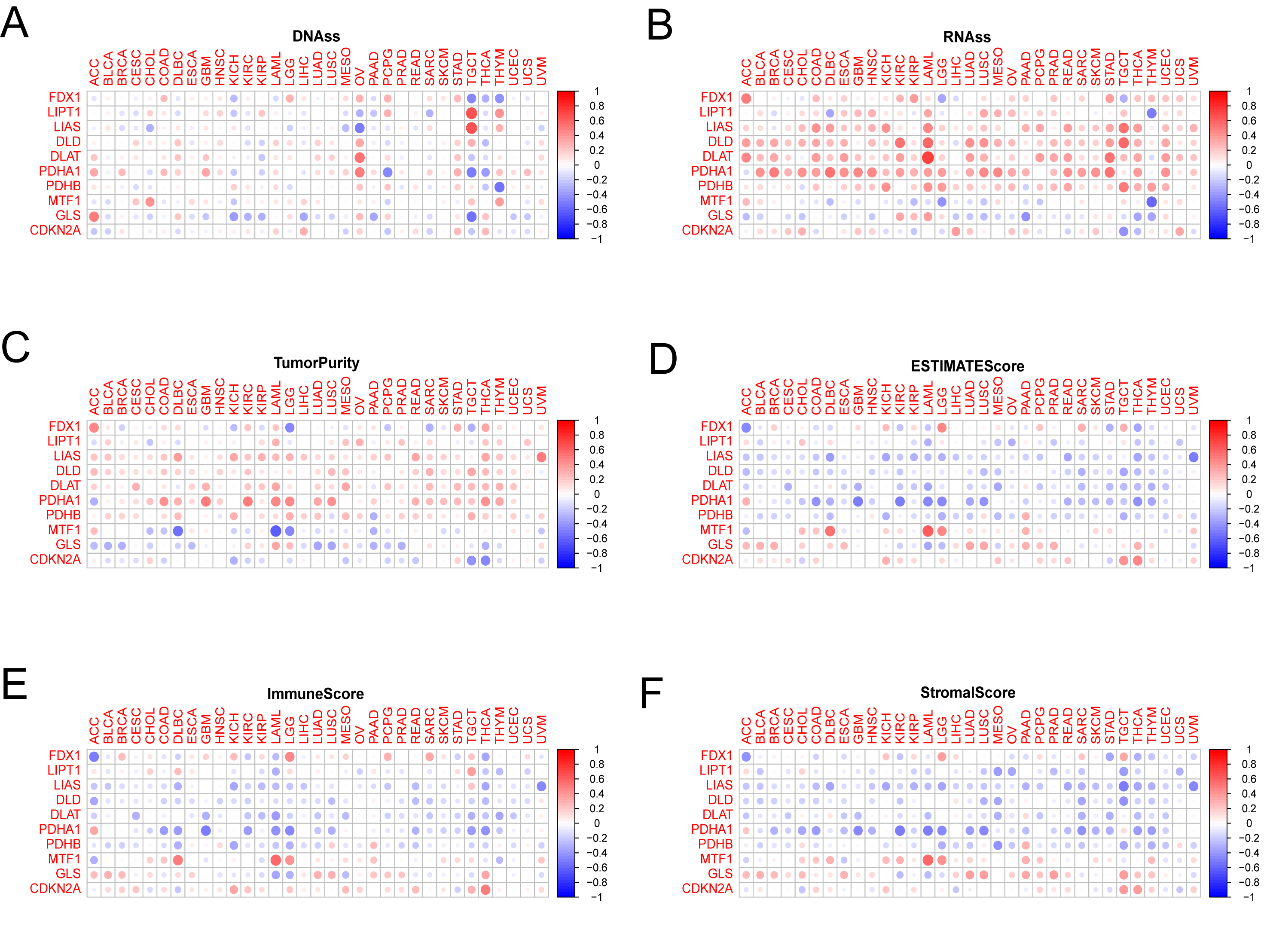
**

**
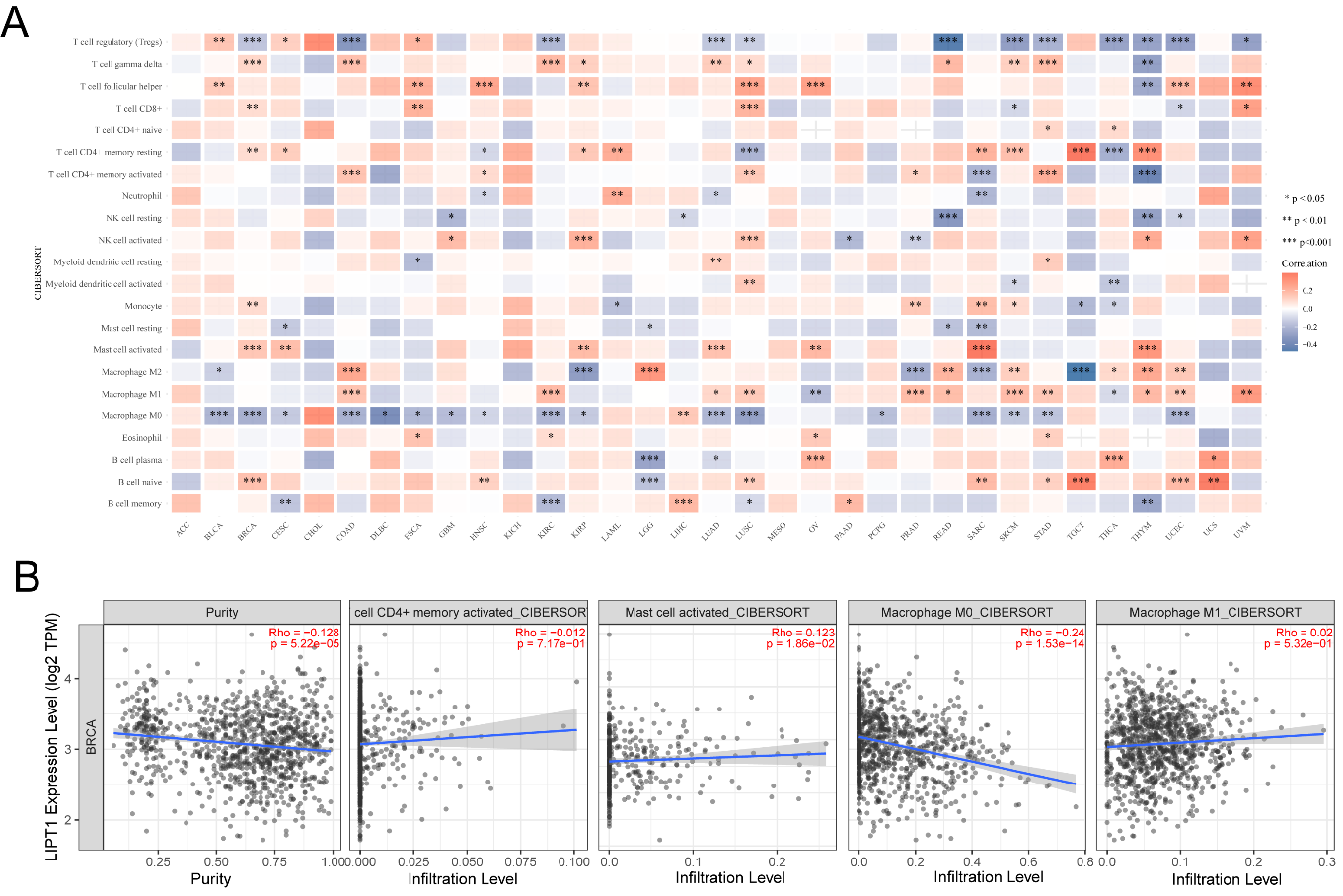
FIGURE S5**

**FIGURE S6**

**
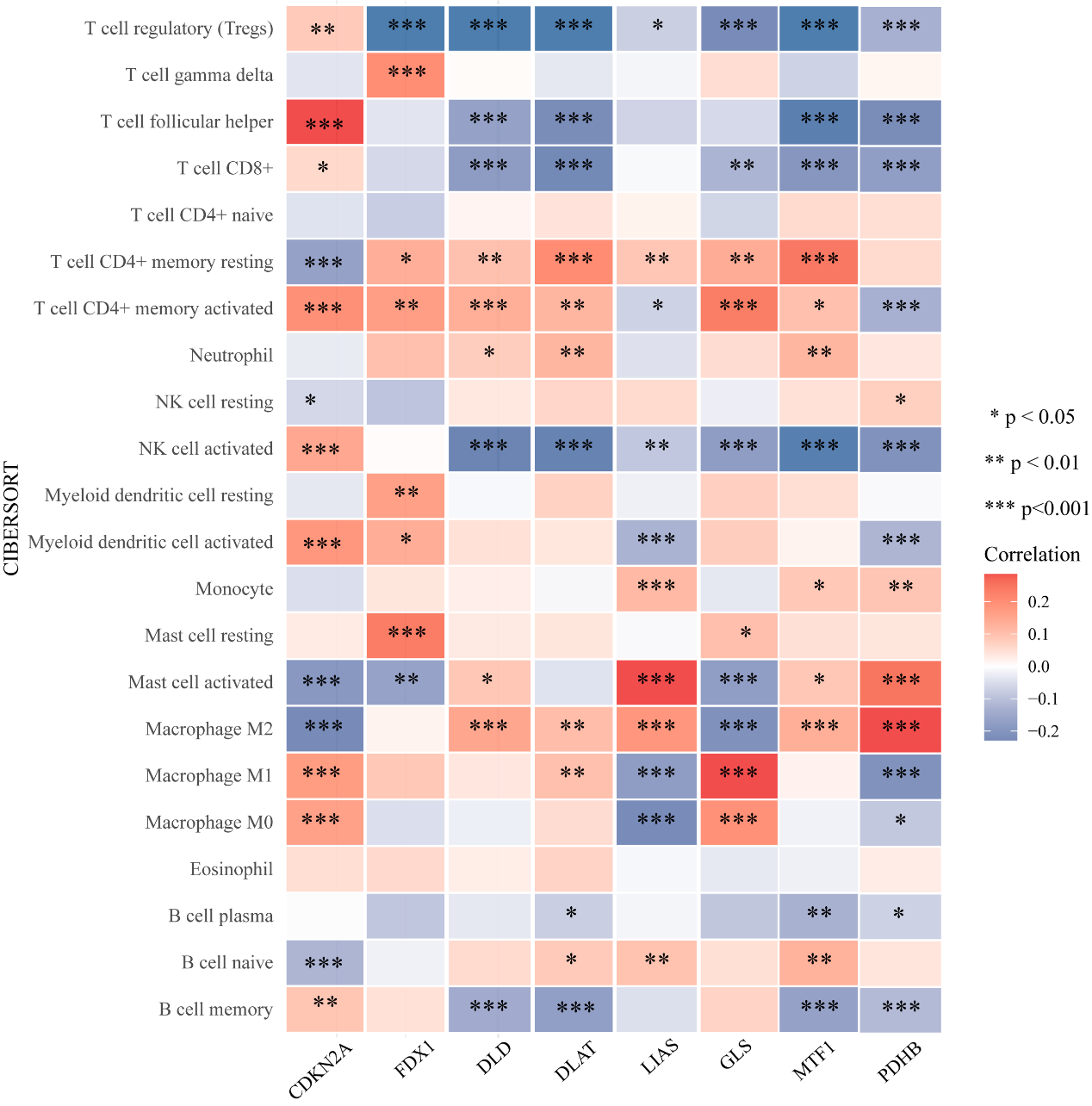
**

**FIGURE S7**

**
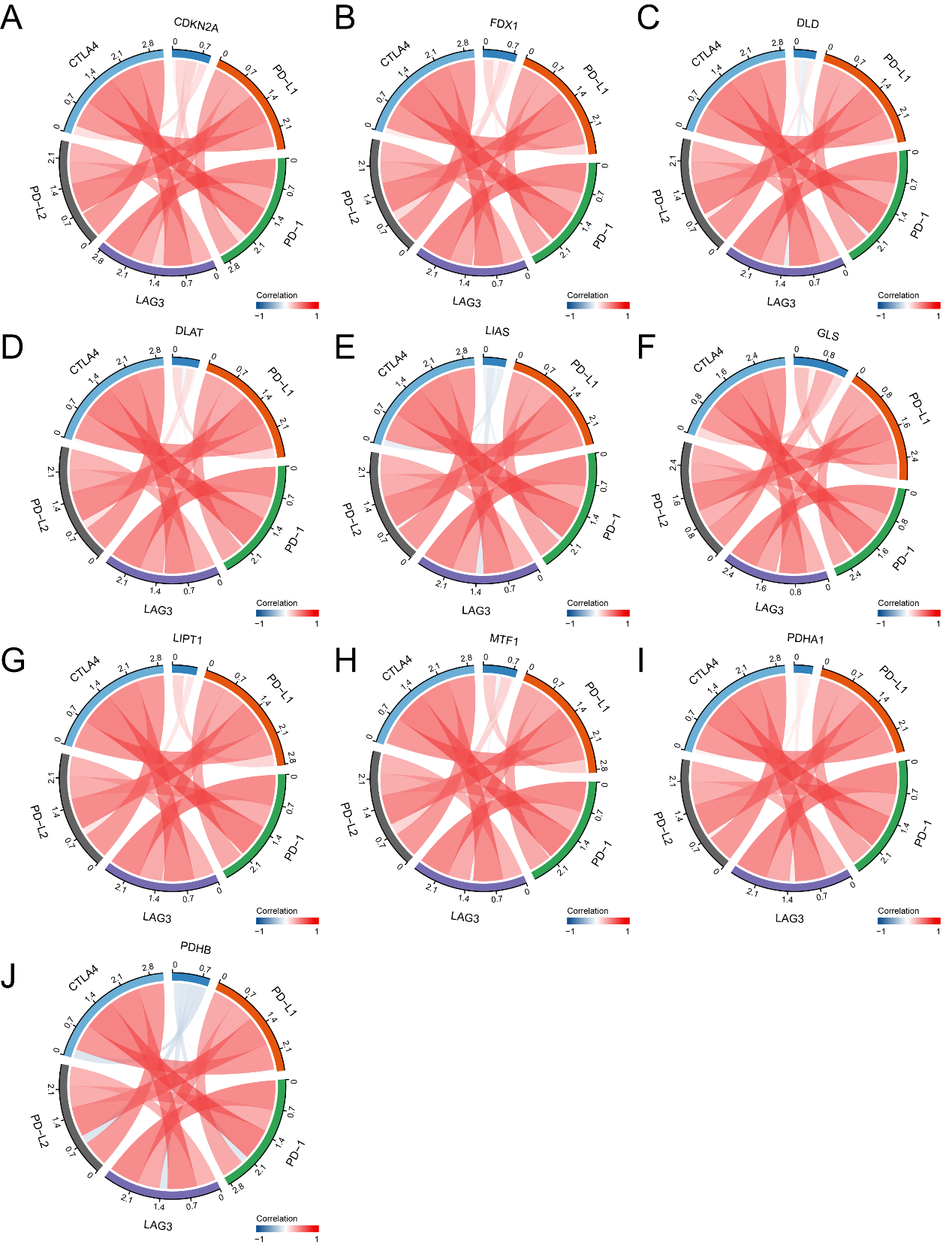
**

**FIGURE S8**

**
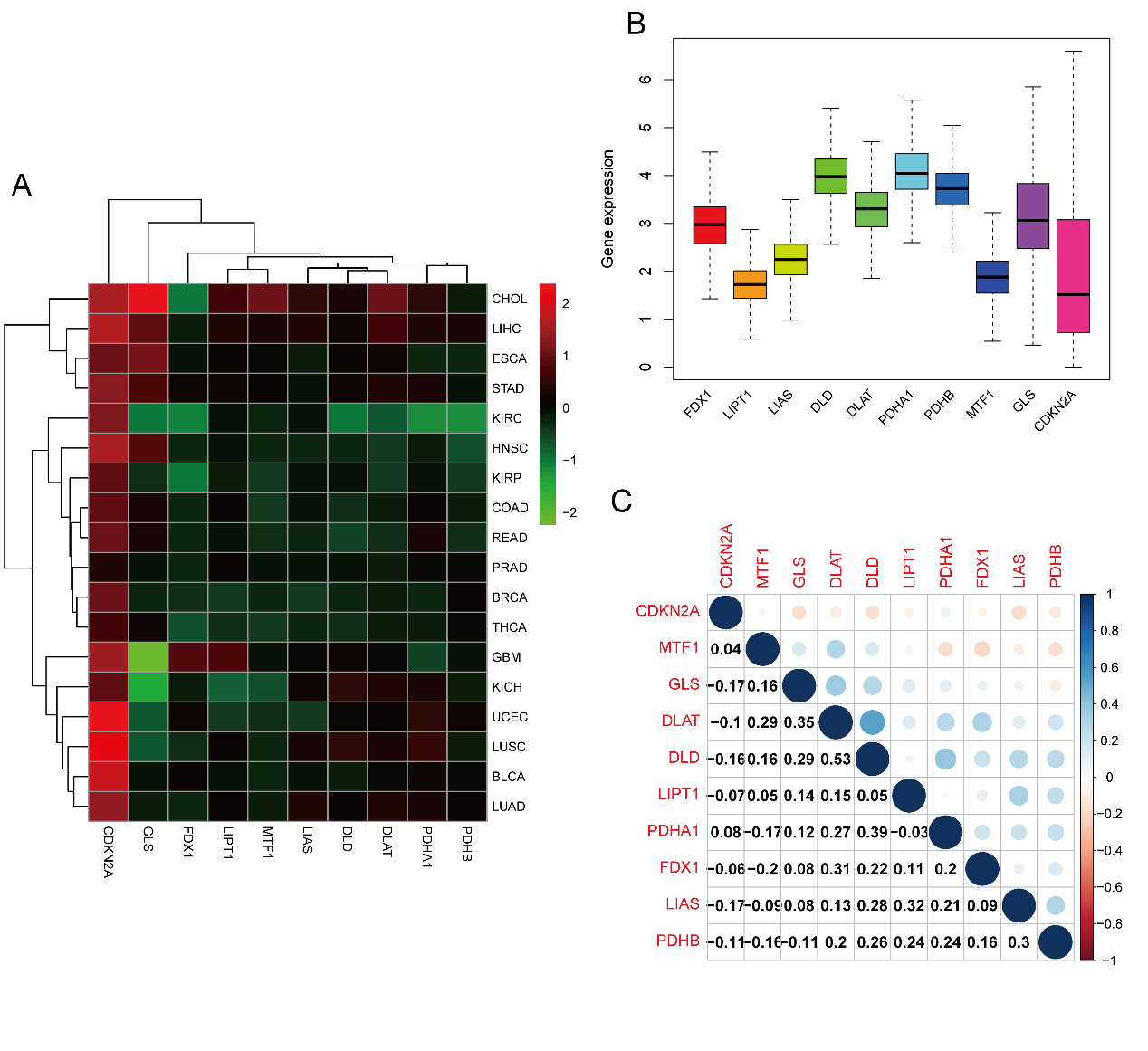
**

**FIGURE S1**

CRGs expression levels in different cancer types and normal tissue. (A) CDKN2A, (B) FDX1, (C) DLD, (D) DLAT, (E) LIAS, (F) GLS, (G) LIPT1, (H) MTF1, (I) PDHA1, (J) PDHB. The red rectangle box are gene expression levels in tumor tissue and the blue rectangle box are normal tissue. * P < 0.05, ** P < 0.01, and *** P < 0.001.

**FIGURE S2**

Correlation analysis between three CRGs expression and early stage survival of BC. (A) CDKN2A, (B) LIPT1, (C) PDHA1. The red lines represent high gene expression, and blue lines represent low gene expression.

**FIGURE S3**

Correlation analysis of PDHA1 expression with clinical characteristics of BC. (A-C) the relationship between PDHA1 expression and age, T stage, N stage from TCGA. (D-F) the relationship between PDHA1 expression and age, T stage, N stage from tissue microarray. (G) Representative images of PDHA1 expression in T stage by immunohistochemistry staining. The scale bar in left images was 100um, and in right images was 50um. The tissue microarray contained 30 BC tissues, including 4 samples with patients over 60 years of age and 26 samples with less than 60 years of age; 11 samples with T1 stage and 19 samples with T2 stage; 14 samples with N0 stage and 16 samples with N1 stage. *P < 0.05, ***P < 0.001.

**FIGURE S4**

Relationship between CRGs expression and tumor microenvironment, Stemness score in pan-cancer. (A, B) CRGs expression is associated with RNAss and DNAss in diverse cancers. Red dots represent a positive correlation and blue dots represent a negative correlation. RNAss, RNA stemness score; DNAss, DNA stemness score. (C) CRGs expression is correlated with tumor purity. (D, E, F) CRGs expression is related to estimate score, immune score, and stromal score in different cancers.

**FIGURE S5**

Correlation analysis of LIPT1 expression with immune cell infiltration analysis in BC. (A) The relationship between LIPT1 expression levels and the infiltration level of immune-related cells. (B) The scatter plots of relationship between LIPT1 expression and infiltration levels of immune-related cells by using TIMER2 database.

**FIGURE S6**

Relationship between eight CRGs expression with immune cell infiltration by CIBERSORT algorithm. * P < 0.05, ** P < 0.01, and *** P < 0.001.

**FIGURE S7**

Correlation between CRGs expression and main immune checkpoint members, including PD-1, PD-L1, PD-L2, LAG3, CTLA4. The red lines represent positive correlation and the blue lines represents negative correlation.

**FIGURE S8**

CRGs differential expression across 18 cancer types and correlation analysis between CRGs expression. (A) Boxplot of CRGs expression in 18 TCGA cancer samples. (B) The heatmap of CRGs differential expression in 18 TCGA cancers. The red boxes indicate that the expression of CRGs is high and the blue boxes indicate that the expression of CRGs is low in correspondence cancer. (C) The correlation analysis of CRGs expression. The blue and red dots demonstrate that CRGs expression level had a negative and positive correlation, respectively.
